# Cognitive levels in testing knowledge in evidence-based medicine: a cross sectional study

**DOI:** 10.1186/s12909-020-02449-y

**Published:** 2021-01-07

**Authors:** Ivan Buljan, Matko Marušić, Ružica Tokalić, Marin Viđak, Tina Poklepović Peričić, Darko Hren, Ana Marušić

**Affiliations:** 1 Department of Research in Biomedicine and Health, University of Split School of Medicine, Šoltanska 2, 21000 Split, Croatia; 2 Department of Psychology, Faculty of Humanities and Social Sciences, University of Split, Split, Croatia

**Keywords:** Medical education, Evidence-based medicine, Learning outcomes

## Abstract

**Background:**

Knowledge assessment in evidence-based medicine (EBM) is usually performed by the measurement of memorised facts, understanding of EBM concepts and application of learned knowledge in familiar situations, all of which are considered lower-level educational objectives. The aim of this study was to assess EBM knowledge both on higher and lower cognitive levels across EBM topics.

**Methods:**

In order to assess knowledge on different EBM topics across learning levels, we created a knowledge test (Six Progressive Levels in Testing – SPLIT instrument), which consists of 36 multiple choice items and measures knowledge in EBM at six cognitive levels (*Remembering*, *Understanding*, *Applying*, *Analysing*, *Evaluating* and *Creating*) and addresses six EBM topics (*Evidence-based practice*, *Internal validity*, *Clinical importance*, *Study design*, *Sources of evidence*, *Diagnostic studies*). Three independent assessors defined the minimum passing score (MPS) for the overall test, based on the first-year course content and educational objectives. The instrument was assessed in a sample of first- (*n* = 119) and third-year medical students (*n* = 70) and EBM experts (*n* = 14).

**Results:**

The MPS was 16 correct answers out of total 36 questions, and was achieved by 21 out of 119 first-year students, 14 out of 70 third-year students and 9 out of 14 EBM experts (χ^2^ = 13.3; *P* < 0.001, with significantly higher proportion of experts passing compared to students). Although experts had the highest scores overall, none of the groups outperformed others on individual cognitive levels, but the experts outperformed students in EBM topics of *Study design* and *Sources of evidence* (*P* = 0.002 and 0.004, respectively, Kruskal-Wallis test). First- and third-year students performed better on specific course topics taught in that study year (*Diagnostic studies* and *Clinical relevance*, respectively).

**Conclusion:**

EBM knowledge of students and experts differ according to the specificities of their education/expertise, but neither group had excellent knowledge in all areas. It may be difficult to develop a knowledge test that includes different EBM topics at different cognitive levels to follow the development of specific and general aspects of EBM knowledge.

## Background

Evidence-based medicine (EBM) is widely accepted as a scientifically supported approach in health care [1]. In 2003, Evidence-Based Health Care Teachers and Developers published the Sicily statement, which recommended that all healthcare professionals should be educated in the field of EBM and follow EBM principles [2]. Subsequently, various learning programs for EBM have been developed and incorporated into medical education, including initiatives such as the Accreditation Council for Graduate Medical Education or CanMeds [3, 4]. However, there is little research focused on the assessment of actual clinical problem-solving using EBM principles, and current evidence does not support the effectiveness of EBM training programs [5].

Although EBM is often emphasized as a skill that needs to be transferred to everyday practice, it is not clear how EBM training leads to the educational objectives developed in theoretical frameworks [6]. Curriculum designers are encouraged to express their educational objectives according to students’ abilities and competencies [6–8]. Those educational objectives, although sometimes differently defined, create sharp differences between the simple memorisation of the material or superficial overview of the information and critical assessment of the acquired information. The assessment is mostly focused on the lowest levels of Miller’s pyramid of assessment of clinical skills, competence and performance (knowledge, competence, performance and action) [7], and thus medical students are still required to memorise materials and facts, without enough of a critical approach [9]. A systematic review of assessment tools in clinical practice demonstrated that the majority were focused on the lower levels of cognition as defined by Bloom’s taxonomy of educational objectives [10]. In our previous study, we compared the three most widely used measures of EBM: the ACE tool [11], Fresno test [12] and Berlin questionnaire [13]. These tests differ in question type, scoring and focus on EBM topics, rendering the results of the student groups or the educational intervention dependent on the choice of the EBM test [14].

Our aim was to assess whether a standardized instrument could be developed to assess knowledge in different EBM topics across different cognitive levels so that the development of knowledge during the medical curriculum could be systematically followed. We developed a knowledge test where six major EBM topics (*Evidence-based practice, Internal validity, Clinical importance, Study design, Sources of evidence, Diagnostic tests*) were assessed at six cognitive learning levels (*Remembering, Understanding, Applying, Analysing, Evaluating, Creating*) [15].

## Methods

### Setting – course description

**Fig. 1:**
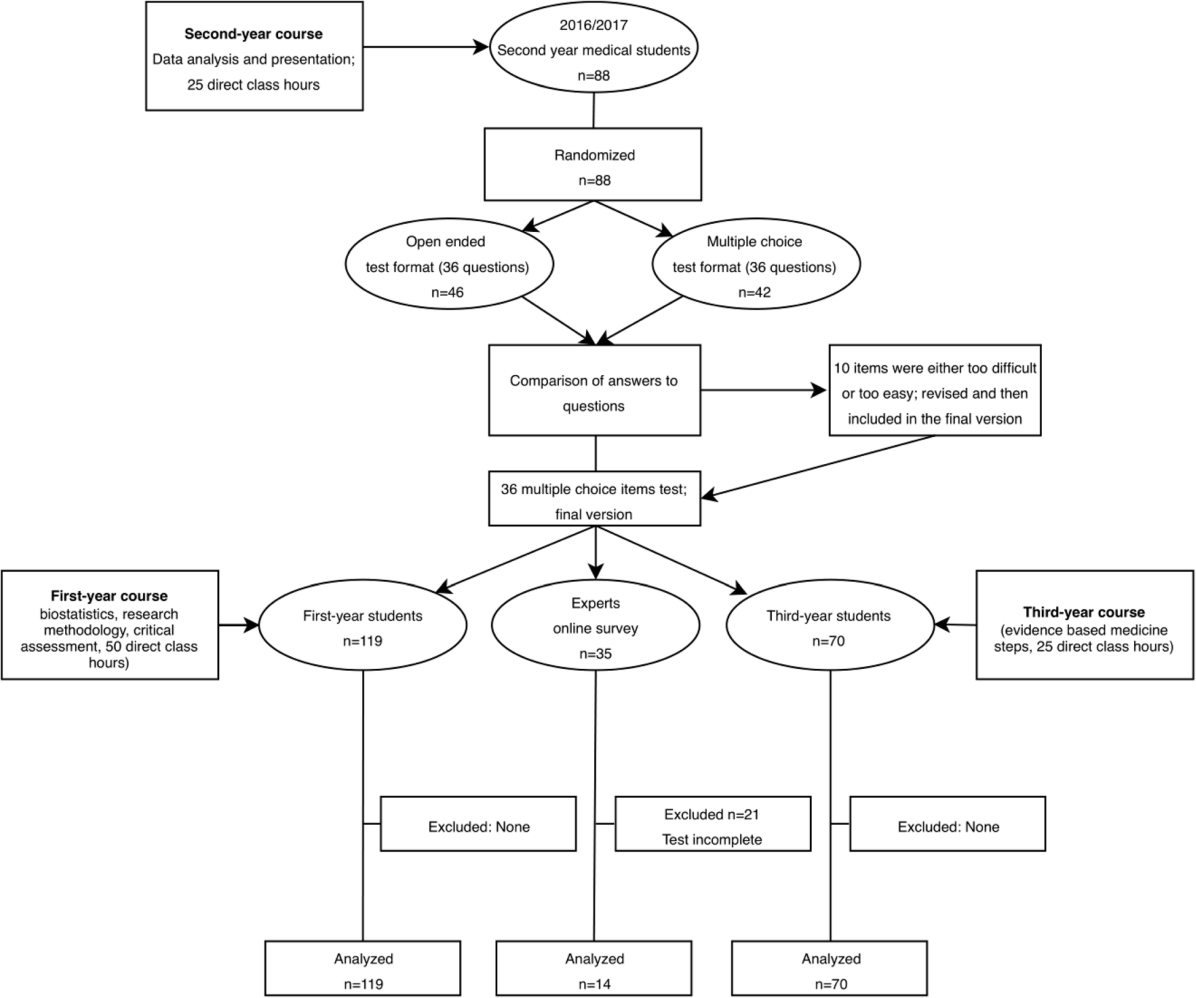
Flowchart of test development and testing groups

At the University of Split School of Medicine (USSM), EBM is a part of a vertically integrated mandatory course in the first 3 years of a six-year integrated medical program that leads to an MD. The first-year course (Research in Biomedicine and Health I) consists of 2 weeks (50 contact hours) of face-to-face lectures, seminars and practical exercises on biostatistics and research methodology. The competencies gained after this first-year course are a basic understanding of research methodology in medicine, critical evaluation of scientific reports, and understanding and application of basic biostatistics [16] (Fig. 1). For the 2016/2017 generation, the topic of the validity of diagnostic study design was introduced, so the outcomes for this generation also included the understanding of the principles of diagnostic studies and evaluation of diagnostic test accuracy.

In the second year (Research in Biomedicine and Health II), students attend 1 week of face-to-face practical exercises (25 contact hours) in which they apply the knowledge gained in the first year to analyse datasets from published research studies and write a brief research report. The expected outcomes are the recognition and application of a suitable statistical test, organization and presentation of research results and critical evaluation of research results (Fig. 1).

In the third year (Research in Biomedicine and Health III with 25 contact hours), students practice the first 3 steps of EBM – formulating PICO questions, searching for evidence and critically evaluating evidence related to specific clinical problems. The expected outcomes are the development and evaluation of a search strategy for a clinical issue, recognition, classification and assessment of the results from Cochrane systematic reviews and meta-analyses, critical appraisal of acquired evidence, and application of quality concepts in health care to solve routine problems in healthcare organization (Fig. 1).

### Participants and procedure

In a cross-sectional study, we first piloted the newly developed instrument with first-year and third-year undergraduate medical students using a pen and paper approach, and with a sample of EBM experts using the online *SurveyMonkey* tool in June 2017.

The student sample (Fig. 1) that consisted of 90 students from the first year and 45 students from the third year of the Croatian medical study programme took the test in Croatian, while 60 students from the first year and 30 students from the third year of the medical study programme in English took the test in English. The test was translated into English by the authors and back-translated by a language expert to ensure the validity of the translation. The students completed the test during their regular Research in Biomedicine and Health classes, but the test was not graded or in any way related to the outcome of the course. The test served as a self-evaluation exercise before the official knowledge test at the end of the course, which was different from the test referred to in this study.

The first participants in the expert sample (Fig. 1) were experts in medical research at the USSM, who passed on information further to other experts in Croatia and abroad, so that the expert sample was created using a snowball approach. The experts received an invitation to participate in research about knowledge assessment in EBM and received no compensation for their participation. The criteria for the qualification as an “expert in EBM” were either: a) being involved in evidence-based medicine as a researcher and/or a teacher, b) having significant previous education in evidence-based medicine, c) having published papers in that area or d) working at university hospitals, universities, research centres or for biomedical journals. We considered the people who satisfied these criteria to be significantly involved in the field and familiar with EBM research concepts and methods. Questions regarding experts’ qualifications were included in the demographic characteristics section on the first page of the *Survey Monkey* questionnaire.

### Development of the instrument

#### Description of the learning levels and EBM topics

The learning levels were defined according to Anderson et al. (2001):

- *Remembering* – ability to recall or retrieve previous learned information;
- *Understanding* – ability to comprehend the meaning, translation and/or interpretation and problems;
- *Applying* – ability to use previously learned knowledge in a new situation;
- *Analysing* – ability to separate material or concepts into component parts so that its organizational structure may be understood;
- *Evaluating* – ability to make judgments about the value of ideas or materials, and/or compare between different ideas or materials; and.
- *Creating* – ability to build a new structure or pattern from diverse elements.
- EBM topics were based on usual topics in EBM courses [9], and the categorization was developed by the authors as follows:
- *Evidence-based practice* – information necessary for the processes in everyday decision-making about the treatment, based on the best available evidence.
- *Internal validity* – knowledge about the appropriateness of the methods used for a specific problem, as well as justified interpretation.
- *Clinical importance* – overall importance of a presented scientific finding for everyday use and practice.
- *Study design* – knowledge about the use of the different study types for different problems.
- *Sources of evidence* – knowledge about the processes of information searching in scientific databases, the use of Boolean operators and defining a search strategy, all of which are recognized by the other EBM measures as one of the steps in Searching the evidence in EBM [11–13].
- *Diagnostic tests* – concepts related to diagnostic tests, the validity of the tests and the choice of a specific diagnostic test.

#### Development of questions for EBM cognitive learning levels

The initial version of the instrument was developed by the authors (IB, MM, AM), with the aim to develop questions that would address different learning levels and EBM topics. The questions were revised by four independent experts (all with a PhD in the field of biomedicine with previous publications and teaching experience in EBM research) who assessed the clarity of the questions, the time needed for their completion and the overall level of test difficulty.

The first version of the instrument was assessed in a group of second-year medical students (during the 2016/2017 academic year). Half of the students randomly received the test containing questions with open-ended answers and half of the students received the same test questions but with multiple choice answers, as a part of their course practical in December 2016. The completion took around 1 h. There was no difference in scores between the open-ended and multiple-choice version, so we used the multiple-choice version because it was simpler to apply and allowed more objective scoring than open-ended answers. Previous research indicated that there is no difference in the assessment of higher cognitive levels when using open-ended or multiple-choice questions [17]. Based on this pilot, we created the final version of the test with 36 questions, with one question for each cognitive learning level and EBM topic. This means that each cognitive level was assessed with six questions, one from each corresponding EBM topic. For example, the *Remembering* cognitive level had six questions assessing the basic recall of the facts, one question focusing on study design, another on information sources, etc.

The result of the test is the total score (the number of correctly answered questions out of total 36 questions), as well as the scores for the individual cognitive levels, where the possible total score was 6, representing the level of knowledge at that level. The only exception in the combination of “six questions from six topics per level” was the final and the highest level, the *Creating* level. The *Creating* level also consisted of six questions, but neither of those six questions could be related to a specific EBM level because questions on that level are very complex and include greater knowledge of different EBM topics for each question, which are difficult to separate (see the test in the Additional file 1).

Since the questions from the *Creating* cognitive level were related to more than a single cognitive level, the scores on the *Creating* level could not be counted for specific EBM topics. Therefore, each EBM topic consisted of five questions that assessed one of the cognitive levels. This means that the total score for individual EBM topics was 5.

For the study, we randomized the questions using online software (www.researchrandomizer.org) in order to prevent possible bias in answering due to the item sequence. The final version of the test (in English) is available in the Additional file 1.

#### Determination of the minimum passing score

In order to define the boundaries in the scoring of the instrument (i.e., the minimum score needed to pass the test for a student from an individual course we used), we used the Minimum Passing Score (MPS) approach (Angoff method) [18]. The Minimum Passing Score can be defined as the minimum score required for the participant to achieve on the test in order to demonstrate a satisfactory level of knowledge [18]. In our case, three assessors with field experience, who did not take part in the creation of the questions (RT, MV, TPP), had to individually and independently choose among the five offered question-answer options, which a student with minimum knowledge about the topic should recognize as correct in order to pass the exam. For example, a student should know that the only correct response to *What is the abbreviation of the checklist used for the assessment of a randomized controlled trial?* is *CONSORT*. A student’s incorrect responses to *Why isn’t the arithmetic mean a representative measure of central tendency in a skewed distribution?* would be *b) Because it is difficult to present graphically* and *c) Because a skewed distribution cannot be mathematically defined*. Based on this assessment, each question was graded depending on the number of the answer options that were not required for the minimum passing in a way that 1.0 (or 100%) was divided by the remaining number of answer options. Specifically, for the question above regarding CONSORT, the score for the question was 1 (or 100%) because it was expected that the student knew the correct answer for minimum passing, which left a 0% chance for guessing. On the other hand, the question about the arithmetic mean was scored as 0.33 (33%) because there were three options that a student did not need to know for minimum passing, which therefore left a 33% chance of guessing the correct answer. We summed the scores for each question to obtain the minimum score needed to pass the test. Particularly, the MPS for the test consisting of four questions, where each question is scored 1 point, which have MPSs of 1, 0.33, 0.33 and 0.33, respectively, would have an overall passing score of 1.99, and therefore the test taker would need to have at least two correct answers to pass the test. Theoretically, the rationale is that for easier questions, MPS should be higher as students should know the answers to basic questions, whereas for the more difficult questions (or higher cognitive levels in this case) MPS should be lower as students are sometimes not required to know some or any of the options for minimum passing [18].

### Sample size calculation

The sample size calculation was made using MedCalc Statistical Software version 17.6 (MedCalc Software bvba, Ostend, Belgium; http://www.medcalc.org; 2017). Using 80% power and 0.05 alpha, we calculated that we needed 11 participants per group in order to obtain the desired difference of 20% (to match a 3 point difference out of 15 points from the ACE test from our previous study [14]) in the scores between groups.

### Statistical methods

Demographic characteristics were presented as frequencies and percentages. Discrimination indexes were used to assess the possibility of differences between participants with greater and lower scores. We calculated the discrimination indexes by comparing the data from the top performing 25% and bottom performing 25% of the participants, first for their overall scores on the test, and then for the scores on individual cognitive levels and EBM topics separately to define how well the question discriminated the participants separately on EBM or cognitive levels [17]. Overall discrimination coefficients, as well as discrimination coefficients for EBM topics and learning levels, the proportion of correct/incorrect answers and MPS were presented as medians (Md) with 95% confidence intervals (CIs), due to the non-normality of the distributions. We calculated the Cronbach’s alpha reliability coefficients for the overall test, for higher and lower cognitive levels, each cognitive level separately and for individual EBM areas, as well as for groups with different levels of expertise. In some cases, due to the low correlations between the items and the small number of items, the reliability coefficients were negative, which indicated poor reliability for the subscale results in that population.

The knowledge scores were presented as medians with confidence intervals. The proportions between groups of participants who achieved the MPS were compared with the chi-square test and pairwise comparisons for proportions (Bonferroni-Holm adjustment method). To address a large number of comparisons, we performed the Bonferroni correction by dividing the 0.05 level with the number of comparisons in the table. The differences between groups with open-ended and multiple-choice questions were compared using the Mann-Whitney non-parametric test and the differences between higher and lower levels were compared by the Wilcoxon non-parametric test. The differences between the first-year students, third-year students and experts were compared using the Kruskal-Wallis non-parametric test with the Dunn post hoc test. All analyses were performed using JASP software, v.0.9. (JASP Team, 2018) and R.v.3.6.3. (R Core Team, 2017).

## Results

In the pilot study, no difference was found between the groups that took the test with open- ended answers (Md = 19, 95% CI = 16 to 19, *n* = 46) and those that took the test with multiple choice answers (Md = 20, 95% CI = 18 to 21, *n* = 42; *P* = 0.722). Ten questions, which were either correctly answered by all participants or remained unanswered by most (90%) of the students, were modified replaced.

In total, 203 participants (69% women) took the test; 119 first-year medical students, 70 third-year students, and 14 experts (representing 40% of 35 experts that started the online test). Most experts had at least 2 years of training in EBM and were involved in EBM as researchers (Table 1).

**Table 1:** Characteristics of experts who participated in the study (*N* = 14)

Variable	No.
Female gender	10
Years of education EBM/research methodology	
Less than one year	1
One year	1
Two years	4
Three years	0
More than three years	8
How many years have you been involved in research?	
One to three years	3
More than three years	11
How many research articles have you published so far?	
Less than five	4
5–10	1
10–20	1
20–40	3
Over 40	5
In which professional capacity are you involved in evidence based medicine?^a^	
As a teacher	8
As a researcher	11
As a practitioner	6
As an editor	4

^a^Multiple answers possible

The test items had low discrimination coefficients when they were calculated for the overall test scores. However, when the discrimination coefficients for each question were calculated based on the scores on an individual EBM topic or cognitive level, the discrimination coefficients increased to an acceptable level (over 0.2) [19] (Table 2).

**Table 2:** Metric characteristics of the instrument questions in overall sample (*N* = 203)

Test question according to cognitive level and EBM topic	Discrimination coefficient^a^	Discrimination coefficient by learning domain^b^	Discrimination coefficient by EBM topic^c^	Proportion of correct answers on overall sample	Proportion of incorrect answers on overall sample	Minimal passing rate (MPR)
*Remembering*						
Evidence-based practice	0.14	0.26	0.22	0.88	0.12	1.00
Internal validity	0.38	0.42	0.52	0.82	0.18	1.00
Clinical importance	0.4	0.54	0.50	0.79	0.21	1.00
Study design	0.4	0.64	0.56	0.70	0.30	0.25
Sources of evidence	0.26	0.38	0.26	0.26	0.74	0.33
Diagnostic studies	0.3	0.64	0.60	0.42	0.58	1.00
Median (95% CI)^d^	0.34 (0.16 to 0.40)	0.48 (0.28 to 0.64)	0.51 (0.23 to 0.59)	0.75 (0.29 to 0.86)	0.26 (0.13 to 0.71)	1.0 (0.27 to 1.0)
*Understanding*						
Evidence-based practice	0.1	0.60	0.34	0.28	0.72	0.33
Internal validity	0.14	0.42	0.40	0.83	0.17	0.33
Clinical importance	0.12	0.16	0.18	0.07	0.93	0.25
Study design	0.2	0.58	0.46	0.68	0.32	0.25
Sources of evidence	0.34	0.5	0.34	0.65	0.35	0.33
Diagnostic studies	0.18	0.54	0.50	0.20	0.80	0.25
Median (95% CI)^d^	0.16 (0.11 to 0.31)	0.52 (0.21 to 0.60)	0.37 (0.21 to 0.49)	0.47 (0.10 to 0.80)	0.53 (0.20 to 0.91)	0.29 (0.25 to 0.33)
*Applying*						
Evidence-based practice	0.48	0.62	0.86	0.54	0.46	0.25
Internal validity	0.24	0.48	0.50	0.36	0.64	0.25
Clinical importance	0.18	0.6	0.72	0.46	0.54	1.00
Study design	0.46	0.68	0.52	0.60	0.40	1.00
Sources of evidence	0.2	0.42	0.20	0.36	0.64	0.20
Diagnostic studies	0.22	0.24	0.38	0.13	0.87	0.20
Median (95% CI)^d^	0.23 (0.18 to 0.48)	0.54 (0.28 to 0.67)	0.51 (0.24 to 0.83)	0.41 (0.18 to 0.59)	0.59 (0.41 to 0.83)	0.25 (0.20 to 1.0)
*Analysing*						
Evidence-based practice	0.16	0.28	0.40	0.19	0.81	0.20
Internal validity	0.54	0.66	0.60	0.63	0.37	1.00
Clinical importance	0.16	0.58	0.74	0.53	0.47	0.20
Study design	0.3	0.58	0.54	0.44	0.56	0.20
Sources of evidence	0.06	0.06	0.06	0.98	0.02	0.50
Diagnostic studies	0.22	0.36	0.44	0.19	0.81	0.20
Median (95% CI)^d^	0.19 (0.08 to 0.49)	0.47 (0.10 to 0.64)	0.49 (0.13 to 0.71)	0.49 (0.19 to 0.91)	0.52 (0.09 to 0.81)	0.20 (0.20 to 0.90)
*Evaluating*						
Evidence-based practice	0.2	0.6	0.70	0.31	0.69	0.25
Internal validity	0.2	0.38	0.58	0.37	0.63	0.33
Clinical importance	0.26	0.34	0.36	0.14	0.86	0.33
Study design	0.22	0.6	0.62	0.56	0.44	1.00
Sources of evidence	0.06	0.08	0.06	0.05	0.95	0.33
Diagnostic studies	0.42	0.72	0.68	0.47	0.53	0.20
Median (95% CI)^d^	0.21 (0.09 to 0.39)	0.49 (0.13 to 0.70)	0.60 (0.12 to 0.70)	0.34 (0.07 to 0.54)	0.66 (0.46 to 0.93)	0.33 (0.21 to 0.87)
*Creating*^e^						
Item 1	−0.06	0.28		0.51	0.49	0.33
Item 2	−0.02	0.38		0.26	0.74	0.20
Item 3	0.06	0.62		0.39	0.61	0.33
Item 4	0.1	0.44		0.26	0.74	0.20
Item 5	0.08	0.54		0.52	0.48	0.33
Item 6	0.1	0.64		0.41	0.59	0.20
Median (95% CI)^d^	0.07 (−0.05 to 0.10)	0.49 (0.30 to 0.64)		0.40 (0.26 to 0.52)	0.60 (0.48 to 0.74)	0.27 (0.20 to 0.33)
Total median (95% CI)^d^	0.20 (0.15 to 0.25)	0.52 (0.41 to 0.59)	0.50 (0.38 to 0.56)	0.43 (0.34 to 0.53)	0.57 (0.47 to 0.66)	0.33 (0.25 to 0.33)

*EBM* Evidence-based medicine, *CI* Confidence interval

^a^Discrimination index ranges from −1 to + 1, and the higher number indicates better discrimination of better and worse performing participants. Discrimination indexes were always calculated based on the comparison of the highest performing 25% and lowest performing 25% participants. Calculated on total sum of all answers (theoretical range 0–36)

^b^Calculated based on the sum of correct answers on questions on corresponding learning domain (theoretical range 0–6)

^c^Calculated based on the sum of correct answers on questions on corresponding EBM topic (theoretical range 0–5)

^d^Medians and corresponding 95% CI were calculated for each domain separately and for overall test scores

^e^For the *Creating* cognitive domain, the questions involved more than one EBM topics. Therefore, questions for the *Creating* domain were arbitrarily labelled based on their sequence in the test

The reliability coefficients were low across all levels and EBM topics for the first-year students (Table 3). For the third-year student population, the reliability coefficients were relatively high for the five-item scales for *Evidence based practice*, *Internal validity* and *Clinical importance*, two EBM topics taught in the third-year EBM course (Table 3). The reliability coefficient for the overall test was the highest for the expert population, where coefficients were high across cognitive levels and for EBM topics related to their expertise (e.g., *Internal validity* and *Sources of evidence*), and low in domains where they were not experts (e.g., *Clinical importance*) (Table 3).

**Table 3:** Reliability coefficients (Cronbach’s alpha) for overall sample and subgroups (*N* = 203)^a^

Variable (n of items)	Overall (*N* = 203)	First-year students (*n* = 119)	Third-year students (*n* = 70)	Experts (*n* = 14)
Overall test (*N* = 36)	0.37	0.32	0.29	0.73
*Lower levels (N = 18)*	0.24	0.19	0.09	0.72
*Higher levels (N = 18)*	0.10	0.05	0.04	0.62
Cognitive levels:				
*Knowledge (N = 6)*	0.24	0.23	0.20	0.62
*Understanding (N = 6)*	0.21	0.08	0.34	0.26
*Application (N = 6)*	0.16	0.22	0.01	0.56
*Analysis (N = 6)*	0.17	0.27	0.12	0.45
*Evaluation (N = 6)*	−0.12	− 0.17	− 0.15	0.47
*Synthesis (N = 6)*	0.01	−0.04	−0.13	0.55
EBM domains:				
*Evidence based practice (N = 5)*	0.06	−0.07	0.30	−0.10
*Internal validity (N = 5)*	0.08	−0.11	0.32	0.40
*Clinical importance (N = 5)*	0.12	0.04	0.27	−0.52
*Study design (N = 5)*	−0.10	− 0.22	−0.10	0.02
*Sources of evidence (N = 5)*	0.10	0.10	−0.05	0.38
*Diagnostic studies (N = 5)*	0.18	0.07	0.09	0.49

^a^Negative coefficients are indicators of low or even negative correlations between items [20]

The median test score for the overall sample was 14 points (95% CI = 3 to 14), but the scores on different cognitive levels varied (Table 4). All participants had higher scores on lower cognitive levels (*Remembering*, *Understanding*, *Applying*) (Md = 9 on 0–18 theoretical range, 95% CI = 9 to 9,) compared to the higher levels (*Analysing, Evaluating, Creating*) (Md = 7, 95% CI = 7 to 7) (*P* < 0.001, Wilcoxon paired samples test). There were no significant differences in the scores on lower or higher cognitive levels separately between the three participant groups (Table 4). For individual EBM topics, experts had significantly higher scores on *Study design* and *Sources of evidence*, third-year students outperformed first-year students on *Clinical importance* and first-year students outperformed third-year students on *Diagnostic studies* (Table 5).

**Table 4:** Scores (median, 95% confidence interval) on cognitive levels by first-year medical students, third-year medical students and experts (*N* = 203)

Cognitive domain level^a^	Overall sample (***N*** = 203)	First-year students (***n*** = 119)	Third-year students (***n*** = 70)	Experts (***n*** = 14)	***P***†
Remembering	4 (4 to 4)	4 (4 to 4)	4 (3 to 4)	5 (3 to 6)	0.151
Understanding	3 (3 to 3)	3 (3 to 3)	2 (2 to 3)	3 (1 to 4)	0.043
Applying	2 (2 to 3)	2 (2 to 3)	3 (2 to 3)	4 (1 to 5)	0.037
Analysing	3 (3 to 3)	3 (3 to 3)	3 (3 to 3)	4 (3 to 4)	0.074
Evaluating	2 (2 to 2)	2 (1 to 2)	2 (1 to 2)	2 (1 to 4)	0.105
Creating	2 (2 to 3)	2 (2 to 3)	3 (2 to 3)	2 (1 to 3)	0.091
Total score (0–36)	14 (13 to 14)	13 (13 to 14)	14 (13 to 14)	18 (11 to 20)	0.042

^a^ Theoretical range for each cognitive level was 0–6

†Kruskal Wallis test with Dunn post hoc comparison. With Bonferroni correction, the level of significance was set to 0.007

**Table 5:** Test scores (median, 95% confidence interval) on EBM topics by the first-year students, third-year students and experts (*N* = 203)

Evidence based medicine topic^a^	Overall sample (***N*** = 203)	First-year students (***n*** = 119)	Third-year students (***n*** = 70)	Experts (***n*** = 14)	***P***†
Evidence based practice	2 (2 to 2)	2 (2 to 3)	2 (1 to 2)	2 (1 to 3)	0.029
Internal validity	3 (3 to 3)	3 (3 to 3)	3 (2 to 3)	4 (2 to 4)	0.104
Clinical importance	2 (2 to 2)	2 (1 to 2)^b^	2 (2 to 3)	2 (1 to 3)	**< 0.001**
Study design	3 (3 to 3)	3 (3 to 3)	3 (3 to 3)	4 (3 to 5)^c^	**0.002**
Sources of evidence	2 (2 to 2)	2 (2 to 2)	2 (2 to 2)	3 (2 to 4)^c^	**0.004**
Diagnostic studies	1 (1 to 1)	2 (1 to 2) ^b^	1 (1 to 1)	2 (0 to 3)	**< 0.001**

†Kruskal Wallis test with Dunn post hoc comparison. Bonferroni correction set the level of significance at 0.007

^a^Theoretical range for each cognitive level was 0–5

^b^Significantly different from the third year

^c^Significantly different than others

The MPS for the test was 15.5 (43.2%), meaning that correct answers to 16 out of 36 test questions was the MPS. In the overall sample, only 50 participants (24.6%) had a total score of 16 or higher. The MPS or higher was achieved by 26 out of 119 first-year students (21.8, 95% CI = 14.3 to 32.0%), 14 out of 70 third-year students (20.0, 95% CI = 10.9 to 33.6%), and 9 out of 14 experts (64.3, 95% CI = 38.8 to 83.7%). A significantly higher proportion of experts achieved the MPS in comparison with first- and third-year students (χ^2^ = 13.3; *P* < 0.001, Bonferroni Holm post-hoc comparison).

## Discussion

The need for new measures of EBM skills which measure both low and high learning levels has been emphasized in several recent systematic reviews, which showed that only a few educational interventions have been assessed by validated instruments. The findings from the systematic reviews further suggested that new and standardized measures of EBM knowledge should be developed; also, there have been few published studies comparing EBM knowledge between experts and students [5, 21, 22]. The assessment of knowledge and application of EBM skills in the appraisal and evaluation of evidence at higher levels rarely occurs due to the lack of standardized instruments [10], because most of the instruments are focused either on EBM knowledge in general or a specific EBM topic and because EBM measures differ in the breadth of the topics that they assess, which can result in biased knowledge assessment [14]. Health professionals who are involved in EBM, through either practice or research, are often expected to know about all aspects of EBM, but their actual knowledge in EBM is rarely tested [23].

To the best of our knowledge, this is the first attempt to systematically test the theory of cognitive domains and its direct association with the learned content on a given subject. We aimed to develop a test that could assess EBM knowledge both across lower and higher cognitive levels and for different EBM topics. However, using a single question per a combination of EBM topics and cognitive levels resulted in low reliability coefficients both for the overall test and for EBM topics or cognitive domain subscales. The low reliability coefficients indicate that the assessment of skills using the current version of the Six Progressive Levels in Testing (SPLIT) test would be imprecise. Our intention was to develop a measure that was simultaneously brief and comprehensive, in order to assess the knowledge of different EBM topics on six cognitive levels. However, due to the high heterogeneity of EBM topics, it was not possible to reliably assess each combination of topics and levels using only a single question per combination. We had two options to increase the reliability of the SPLIT knowledge test. We could either eliminate all the items that correlated poorly with the total score in order to increase the reliability of the test, but the result would be a very narrow measure of EBM, in which we would not be able to examine all EBM topics across all cognitive levels. On the other hand, increasing the number of items per EBM-cognitive level combination would increase the reliability of the test by omitting questions that poorly correlate with the overall score. However, this would make the test longer and time-consuming, possibly affecting the motivation of the test takers. In the current version of the test, 60% of the expert sample did not complete the test and we can presume that the response rate would be even lower if the test was longer. Also, although we did not capture the differences between the groups on different cognitive levels, we think that the lack of the ability of the test to discriminate between poor and good performers was not the underlying reason because discrimination indices for individual levels were very high. Therefore, if the differences between groups really existed, we would have captured them in our analysis. These issues should be taken into consideration when developing any EBM knowledge test that includes cognitive levels. Possible solutions include focusing on a single, specific EBM topic across all six cognitive levels, where several highly correlated questions could be developed to increase precision. On the other hand, if the aim is to assess the knowledge of different EBM topics, then it is possible to address a specific cognitive level (e.g., “high” but not “low” cognitive levels). Clearly, the decision would depend on the population assessed, EBM topic relevance and desired educational outcomes.

Medical professionals and medical students, regardless of their level of professional expertise, scored higher on lower cognitive levels in our test, but not on higher cognitive levels. The students and experts also differed in their command of six different EBM topics. The students had higher scores on the EBM topics that were taught in their current course, which may indicate that EBM knowledge also depends on the context in which EBM is used in everyday situations or in which it was recently learned. These results show that most of the participants scored better on lower and less demanding cognitive domains, and that only a few of them achieved better scores on higher domains. Individual student groups in our study were better in the EBM topics that they had recently studied, whereas experts may have had better scores at higher levels only for aspects related to their everyday work, but not in others. For example, a possible reason for first-year medical students’ achievement of higher scores compared to the third-year medical students on the EBM topic *Diagnostic studies* could be that teaching on diagnostic studies had been implemented in the EBM course in the first year as a standalone teaching objective in the 2016/2017 academic year. Previously, the inclusion of diagnostic studies as a topic constituted a small part of a general lesson on study design. Third-year medical students had significantly higher scores compared to first-year medical students on *Clinical importance*, probably because the third-year course focuses on the clinical relevance of medical interventions and their effects [16]. However, there was no difference between the student groups in any of the cognitive levels, and both groups followed similar patterns, scoring higher on lower cognitive levels and lower on the more complex levels. It appears that students’ knowledge acquired during the first year does not decrease over time, because third-year medical students’ knowledge in EBM topics was not inferior compared to first-year medical students. It is likely that without further repetition of knowledge in specific EBM topics (e.g., actual research planning and conducting during the clinical years of medical education), further improvement in the accomplishment of educational objectives is difficult to achieve, and the most likely result is the stagnation of the knowledge level [24].

Most of our experts reported that they worked in EBM research rather than practice, which may explain why they scored the highest on the EBM topics related to research (*Study design*, *Sources of information*) and lower on topics related to clinical practice (*Diagnostic studies* and *Clinical importance*). On top of that, the lack of increase in test scores in third-year students over first-year students implies that people only recall clearly the information that they have just learned or use regularly. This also seems to be true for the experts, who also scored better on topics related to their specific expertise. Our findings evoke an important question about whether similar results could be expected in other areas of medicine (e.g., anatomy, physiology and the clinical sciences) if knowledge is tested by carefully and consistently designed tests addressing all six cognitive level, or even among EBM experts with specialized expertise in different medical areas. Our finding that the group of internationally recognized experts showed the same imperfections (“holes”) in their knowledge as students is concerning in regard to contemporary practice, expectations and conclusions of testing specific knowledge in general.

These questions were developed according to the learning levels from Bloom’s topology and were piloted with experts in education and EBM. It is possible that the participants in our study did not deal with higher cognitive level problems in EBM, including the experts, which may have led to the small difference between the groups at the higher level questions in the test. The low results on the SPLIT test were mostly due to wrong answers to higher cognitive level questions than in other instruments, which are often perceived as difficult [10]. In our recent study comparing three EBM tests that are commonly used for medical students and are focused on the lower cognitive domains (knowledge/recognition, understanding and application), students from our medical school scored about 50%, similar to other reported results for medical students around the world [11]. EBM experts were systematically (but not significantly) better compared to students on the individual cognitive levels, so that their total score was significantly higher on the overall test. First-year students and experts had higher scores for the *Diagnostic studies* EBM topic compared to third-year students, and third-year students outperformed first-year students on the *Clinical importance* topic. Experts outperformed students on *Study design* and *Information sources* topics. Regarding the questions from different EBM topics, experts scored no different from students on questions about *Evidence-based practice* or *Internal validity* of studies, despite their greater overall results. These findings suggest that the knowledge of scientific methodology in medicine is rather complex, and the EBM experts are specialized only in certain but not all topics. The conclusion to be drawn is that none of the groups had excellent overall knowledge in EBM, but that some participants had greater knowledge in certain topics, and lesser in others. To improve EBM knowledge, EBM should be taught and assessed continually, and the outcomes of learning processes should include outcomes broader than knowledge [25].

Our study must be interpreted in light of several limitations. The instrument we developed was tested in a single institution and future research should explore whether the results are generalizable to other populations and educational settings. The sample size in the expert sample was small, and they had the option to take the survey online, unlike the student sample. However, this was the only way that we could engage experts from different institutions to take the test. More than half of the experts who entered the survey left it after providing their demographic data (Fig. 1). Perhaps the professionals who completed the test were active in and/or enthusiastic about EBM, or that those who started the test but did not complete it realised that they lacked knowledge to answer the questions and did not want to continue. The two groups of students took the test in two different languages, but we performed back-translation before the application of the test to ensure the validity of the content. Additionally, all questions were in a multiple-choice format, even though higher cognitive levels are usually examined using open-ended questions [17]. However, in order to standardize the content and questions and to allow consistent scoring, we chose to develop a multiple-choice test based on evidence from previous studies that multiple-choice questions are not inferior to open-ended questions [20], which we confirmed in the pilot testing of this study.

## Conclusion

An assessment of EBM knowledge should be performed both on higher and lower cognitive levels and the extent of testing of different EBM topics must be attuned to the educational goals and materials specific for educational settings. The SPLIT knowledge test developed in this study demonstrated the advantages of assessing different EBM topics equally, thus reducing possible bias in EBM knowledge assessment, but resulted in low precision of the knowledge assessment. For a more precise assessment, there should be a greater number of items per each specific domain and cognitive level. Use of separate assessment measures, which continually progress in cognitive levels, would assess knowledge at different learning points. The progression in cognitive levels can be used to assess improvements in participants’ cognitive development during EBM studies. Using the test that is a combination of questions addressing different cognitive levels and EBM topics may help EBM trainers to follow the progression of knowledge acquisition in specific populations and specific educational interventions.

## Supplementary Information

**Additional file 1.**

## Data Availability

The datasets used and analysed during the current study are available from the corresponding author upon reasonable request.

## Abbreviations

EBM: Evidence-based medicine
SPLIT instrument: Six Progressive Levels in Testing instrument
MPS: Minimum passing score
PICO: Patient, Intervention, Comparison, Outcome
