# Supplementary material for: Cognitive levels in testing knowledge in evidence-based medicine: a cross sectional study
Source: BMC Med Educ. 2021 Jan 7;21:25. doi: 10.1186/s12909-020-02449-y (PMC7791849; doi:10.1186/s12909-020-02449-y)
Supplement: Supplementary file 1 — Additional file 1. [file 12909_2020_2449_MOESM1_ESM.docx]

**Six progressive levels in testing of evidence-based medicine (SPLIT) instrument**

**EB-KN. To increase study power, one should increase:**

1. Sample size
2. P value
3. Statistical test power
4. Internal validity of research
5. Specificity

**IV-UN. Why isn't arithmetic mean a representative measure of central tendency in a skewed distribution?**

1. Because a skewed distribution does not have a measure of central tendency.
2. Because it is difficult to present graphically.
3. Because skewed distribution cannot be mathematically defined.
4. Because a single extreme result can significantly alter the mean.
5. Because calculating arithmetic mean includes squaring, which in case of skewed distribution disproportionally increases extreme values.

**CI.AN. What is the efficacy of treatment whose NNT is 5 (95% confidence interval 3-43)?**

1. Small, because NNT is small and confidence interval includes 5.
2. Large, because NNT is small and confidence interval includes 5.
3. Small, because NNT is small and confidence interval is large.
4. Large, because both NNT and confidence interval are large.
5. Small, because NNT is large and confidence interval is small.

**Read summaries of the next two studies carefully and answer the questions EB-EV and IV-EV.**

**Study 1**

Association between daydreaming while driving and risk of a traffic accident in which driver holds the cilpability has been studied. A case-control study was conducted, including 955 drivers who were hurt in a traffic accident. Intensive daydreaming was found to be related to the liability for the traffic accident (17% in accidents in which drivers considered themselves responsible, and 9% in accidents where drivers thought they were not directly responsible), OR=2.12, 95% CI 1.37-3.28).

**Study 2**

The goal of the study was to investigate the relation between migraine and sudden cardiovascular events in women. A cohort of women was followed prospectively from 1989 to 2011. 90% of 115.541 initially included participants finished the study. Outcome was a „big “cardiovascular event: heart attack (myocardial infarction), a stroke (cerebrovascular insult or accident) or some other form of lethal cardiovascular morbidity. Migraine was related both to the increased risk of a big cardiovascular event (relative risk 1.50, 95% CI 1.33-1.69) and increased risk of death by cardiovascular morbidity (relative risk 1.79, 95% CI 1.33-2.32). Study results show there is a consistent relationship between migraine and cardiovascular disease.

**EB-EV. Mark the correct statement of the following:**

1. Study 1 has larger power and is more applicable.

B. Study 2 has larger power, but is less applicable.

C. Study 1 has more confounding factors.

D. Study 2 has smaller power and smaller applicability.

E. Both studies have equal power and applicability.

**IV-EV. Mark the correct statement of the following:**

1. Study 2 had larger power because of the larger sample size.
2. Study 2 shows that migraines have an influence on sudden cardiovascular events.
3. Study 1 was prospective.
4. Study 1 shows daydreaming causes traffic accidents.
5. Confidence interval of both studies are too wide.

**SD-EV. Why does a randomised controlled trial (RCT) provide better quality of evidence than a cohort study?**

1. RCT has a control group, and a cohort study does not.
2. RCT has balanced group sizes, and a cohort study does not.
3. RCT is cheaper than a cohort study.
4. RCT is shorter in duration than a cohort study.
5. RCT controls exposure to investigated factor, and a cohort study does not.

**IS-KN. The guideline for reporting cohort study results is called:**

1. COCHRANE.
2. PRISMA.
3. STARD.
4. CONSORT.
5. STROBE.

**IS-UN. You want to search databases for information on breast cancer. Searching the key word „breast“ retrieved 4200 articles, and key word „cancer“ retrieved 34150 articles. Which of the following statements is correct:**

1. Using those two key words and the operator AND will retrieve 38150 articles.
2. By using the operator OR to combine „breast“ and „cancer“ will retrieve at least 4200 articles.
3. Search strategy „breast NOT cancer“ will retrieve more than 4200 articles.
4. Search strategy „cancer NOT breast“ will retrieve 34150 articles.
5. By using the operator OR you will get fewer than 4200 articles.

**DS-EV. It is better to have high sensitivity and low specificity of a diagnostic test, than a low sensitivity and high specificity, if:**

1. The test is very cheap.
2. The disease is very dangerous.
3. Additional diagnostic procedures are very aggressive.
4. The disease is very rare.
5. The test is time consuming.

**Choose the correct combination of answers that fits the research plan. Each answer is made up of six parts of research plan: hypothesis, study design, inclusion criteria, exclusion criteria, main outcome measure and one potentially confounding factor.**

**S1. You want to know if eating ice cream too quickly causes headache.**

| **Answer** | **Hypothesis** | **Type of study design** | **Exclusion criteria** | **Outcome measure** | **Confounding factor** |
| --- | --- | --- | --- | --- | --- |
| A | Eating ice cream too quickly causes headaches. | Cross-over trial | Subjects with frequent migraines | Incidence of headache | Headache localization |
| B | Eating ice cream too quickly causes headaches. | Qualitative study | Subjects with frequent migraines | Incidence of headache | Headache localization |
| C | Eating ice cream too quickly causes headaches. | Cross-over trial | Subjects with frequent migraines | Incidence of headache | Speed of eating ice cream |
| D | Eating ice cream too quickly causes headaches. | Qualitative study | Subjects with frequent migraines | Incidence of headache | Speed of eating ice cream |
| E | Eating ice cream too quickly causes headaches. | Cross-over trial | Subjects with frequent migraines | Incidence of headache | Headache localization |

**IV-KN. Prevalence of disease in a population is best determined by:**

A. A randomized controlled trial (RCT).

B. A cohort study.

C. A cross-sectional study.

D. A qualitative study.

E. A retrospective study.

**IV-AN. Among given studies, choose one with design that is at the top of the pyramid of clinical relevance:**

1. To investigate attitudes towards mental health patients, the researchers conducted a phone survey on a sample of Croatian citizens, choosing the participants randomly from telephone directory.
2. In order to closely explore the relation between zinc intake and prostate cancer, the researchers compared a group of prostate cancer patients with a group of urologic patients without cancer and assessed differences in their zinc levels.
3. To investigate which therapies help in alopecia areata treatment, the researchers analysed 17 randomized controlled trials of alopecia treatment.
4. To check the efficacy of new alcohol rehabilitation treatment, participants were randomized into two groups, one that was treated with a new anxiety medication and a second group that that was given older and well known anxiety medication. Main outcome measure was relapse, i.e. dropping out of rehabilitation programme.
5. To determine if lower cardiovascular fitness is associated with sudden depression, 12 prospective cohort studies were analysed.

**SD-KN. Randomized controlled trial (RCT) outcome measures are all of the following except:**

1. Incidence.
2. Prevalence.
3. Event rate.
4. NNT.
5. Absolute risk.

**EB-AP. Prevalence of headache in one year on a sample of Split-Dalmatia County citizens was measured, and these are the results:**

**
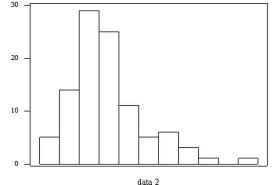
**

No. of participants

No. of headaches per year

| Arithmetic mean | 16.8 |
| --- | --- |
| Median | 12 |
| Standard deviation | 2.79 |
| Interquartile range | 6 |
| Total range | 2-35 |

**Which measures should be used to describe the sample?**

1. Arithmetic mean and standard deviation (M=16.8 i SD=2.79).
2. Median and interquartile range (Md=12 i IQR=6).
3. Arithmetic mean and total range (M=16.8 and total range 2-35).
4. All of 5 listed measures, depending on statistical test being used.
5. Median and standard deviation (Md=12 i SD=2.79).

**IS-AN. Which part of a scientific article does the next sentence belong to: „Number of prepared questionnaires was defined according to hospital employee list (total of 750 physicians)“:**

1. Introduction.
2. Methods.
3. Results.
4. Discussion.
5. Appendix.

**DS-KN. The proportion of true negative results of a diagnostic test in the total number of healthy participants is called:**

1. Positive predictive value.
2. Precision.
3. Negative predictive value.
4. Specificity.
5. Sensitivity.

**IS-EV. Two systematic reviews were produced. The first one was conducted in 2009, and included 43 randomized controlled trials that tested efficacy of cognitive behavioural therapy in treating anxiety attacks. An update was not conducted. The second SR was performed in 2016, and included 10 RCTs that investigated influence of working with computers and eyesight worsening. Which of the following statements is correct:**

1. To assess the reliability of the studies, it is necessary to look at their conclusions.
2. First systematic review is more reliable because it includes RCTs.
3. Second systematic review is more reliable because it is more recent.
4. First study is more reliable because it includes more studies.
5. First study is more reliable because it includes more studies and more randomized controlled trials.

**CI-AP. A new treatment for social anxiety was investigated and it provided normal everyday functioning in 20% of the patients, compared to 10% of the patients who were treated by standard treatment. How many patients should be included in the new program to assure that one of them achieves normal everyday functioning?**

A. 2.

B. 5.

C. 20.

D. 10.

E. 15.

**S3. Choose a research plan that would provide you with answers on weekly alcohol consumption and its long-term consequences.**

| **Answer** | **Goal of the study** | **Study design** | **Exclusion criteria** | **Outcome measure** | **Confounding factor** |
| --- | --- | --- | --- | --- | --- |
| A | To investigate long term consequences of alcohol consumption on a weekly basis. | Randomized controlled trial | Subjects who drink alcohol several times a week. | Incidence of subjects with side-effects | Subjects who drink different quantities of alcoholic beverages. |
| B | To investigate long term consequences of alcohol consumption on a weekly basis. | Cohort study | Subjects who don't drink alcohol. | Incidence of subjects with side-effects | Subjects who drink various alcoholic beverages. |
| C | To investigate long term consequences of alcohol consumption on a weekly basis. | Case-control study | Subjects who drink various alcoholic beverages. | Odds ratio that measures association between weekly consumption of alcohol and its long term consequences | Subjects who drink different quantities of alcoholic beverages. |
| D | To investigate long term consequences of alcohol consumption on a weekly basis. | Cohort study | Subjects who drink alcohol several times a week. | Incidence of subjects with side-effects | Subjects who drink different quantities of alcoholic beverages. |
| E | There are long term consequences of alcohol consumption on a weekly basis. | Cross-sectional study | Subjects who drink various alcoholic beverages. | Prevalence of subjects with side-effects | Subjects who drink various alcoholic beverages. |

**CI-UN. A study of relation between consumption of black tea and weight loss in overweight participants gave the following results: group that consumed black tea for six months lost an average of 328 g (95% CI 125 – 922 g), and the group that did not lost an average of 121 g (95% CI 50 – 211 g). What conclusions can you draw from this research?**

1. Black tea helped in reducing bodyweight for an average of 207 g.
2. Black tea helped in reducing bodyweight, but the result is not statistically significant.
3. Black tea helped in reducing bodyweight and the result is statistically significant.
4. We cannot make conclusions about efficacy of black tea from available data.
5. Black tea did not help in reducing bodyweight.

**S5. You want to investigate the efficacy of corticosteroids in the treatment of chronic foot dermatitis in comparison to antibiotics, which are considered to be the golden standard.**

| **Answer** | **Study**  **design** | **Inclusion criteria** | **Exclusion criteria** | **Outcome measure** | **Confounding factor** |
| --- | --- | --- | --- | --- | --- |
| A | RCT | Subjects with chronic foot dermatitis | Subjects who have psoriasis | NNT for corticosteroids | Subjects using both treatments |
| B | Cohort study | Subjects with chronic foot dermatitis | Subjects who have a fungal infection | Relative risk reduction | Time which passes and aids treatment |
| C | Crossover trial | Subjects who agreed to participate in the research | Subjects who have a fungal infection | NNT for corticosteroids | Effects of the first treatment to the current one |
| D | Systematic review of RCTs | Subjects with dermatitis | Subjects who have a fungal infection | Comparison of NNT for corticosteroids and for antibiotics | Possibility of a very few studies available |
| E | Systematic review of cohort studies | Subjects who do not have foot dermatitis | Subjects who have psoriasis | Absolute risk | Lack of a control group |

**SD-UN. While investigating a possible connection between marijuana smoking and lung cancer, it was found that the number of participants who smoked marijuana was significantly higher in the group who had lung cancer than the healthy control group (OR=1.3, 95% CI 1.1-1.7). Correct interpretation of this result is:**

1. Subjects who smoke marijuana are 10-70% less likely to get lung cancer.
2. Subjects who smoke marijuana are 30% more likely to get lung cancer.
3. Subjects who smoke marijuana are 30% less likely to get lung cancer.
4. There is no association between marijuana smoking and lung cancer.
5. No conclusion can be made on the association between marijuana smoking and lung cancer.

**EB-UN. While comparing the efficacy of drug A and drug B, it was found that drug B is significantly more efficient than drug A (P=0.017). If the study power was 0.8, what can you conclude from that information?**

1. That alpha value is 0.8.
2. That the sample is made of 4/5 of the population.
3. That the study includes 80% of the participants.
4. That the difference found between groups is significant at 80% probability level.
5. That there is 80% probability of finding a difference between groups.

**SD-AP. A conclusion that participants exposed to a factor A had lower incidence of disease was made out of:**

1. A cross-sectional study.
2. A cohort study.
3. A diagnostic study.
4. A physiological study.
5. A case-control study.

**DS-UN. Likelihood ratio for a test result was 12.4. We can conclude that:**

1. The subject has disease.
2. The subject does not have disease.
3. The test is not sensitive.
4. The test has high specificity.
5. The test finds disease in every 12th subject.

**CI-KN. The result of a case-control study is expressed as:**

1. Odds ratio.
2. Prevalence.
3. Absolute risk reduction.
4. Relative risk.
5. Positive predictive value.

**S6. Diagnostic test for colon cancer is very expensive and you want to develop a new one that would cost less. Parts of your research will be as follows:**

| **Answer** | **Hypothesis** | **Study design** | **Inclusion criteria** | **Exclusion criteria** | **Outcome measure** | **Possible bias** |
| --- | --- | --- | --- | --- | --- | --- |
| A. | There will be no difference between tests in the proportion of true results | Cross-sectional study | Subjects who want to find out if they have colon cancer | None | Specificity and sensitivity of the new test | Not all subjects are tested with the golden standard |
| B. | Is there a difference between tests in the proportion of true results? | Cohort study | Subjects who have colon cancer | Subjects who do not have colon cancer | Number of subjects who develop colon cancer | Big attrition rate |
| C. | Is there a difference between tests in the proportion of true results? | RCT | Subjects who have colon cancer | Subjects who do not have colon cancer | Number of subjects who do not develop colon cancer | Small sample size |
| D. | There will be no difference between tests in the proportion of true results | Cohort study | Subjects who do not have colon cancer | Subjects who have colon cancer | Number of subjects who do not develop colon cancer | Not all subjects are tested with the golden standard |
| E. | New test will be better at diagnosis compared to the golden standard | Cross-sectional study | Subjects who apply for research | Subjects who have colon cancer | Specificity and sensitivity | Difficult to randomize |

**S2. Your patient wants to know how likely it is that he has prostate cancer. The patient does not suffer from any medical condition. You search PubMed to find research that would provide you with an answer to that question. What combination of search terms would you use?**

| **Answer** | **Goal of the study** | **Study design** | **Exclusion criteria** | **Outcome measure** | **Confounding factor** |
| --- | --- | --- | --- | --- | --- |
| A | Patient does not have prostate cancer. | Cross-sectional study | Subjects who have several types of cancer. | Incidence of subjects with prostate cancer | Country of research |
| B | Patient has prostate cancer. | Systematic review of RCTs | Subjects in good health. | Incidence of subjects with prostate cancer | Country of research |
| C | To check the likelihood of your patient not having prostate cancer. | Systematic review of cross-sectional studies | Subjects who have prostate cancer. | Prevalence of subjects with prostate cancer | Sample size |
| D | To check the likelihood of your patient having prostate cancer. | Systematic review of cohort studies | Subjects who have several types of cancer. | Incidence of subjects with prostate cancer | Sample size |
| E | To check the likelihood of your patient having prostate cancer. | Cross-sectional study | Subjects who have several types of cancer. | Prevalence of subjects with prostate cancer | Country of research |

**IV-AP. A physician wants to investigate differences in rates and numbers of metastasis in all of the hospital lung cancer patients, depending on their cancer stage (I.-IV.). The sample in such research will be:**

1. Convenience sample.
2. A random sample.
3. A systematic sample.
4. A stratified sample.
5. A cluster sample.

**SS-AN. In cohort studies, what is the advantage of prospective compared to historical prospective study design?**

1. A prospective study is cheaper.
2. In a prospective study participants can be randomized.
3. A prospective study has the possibility of analysing adverse events, and a historical study does not.
4. A prospective study requires smaller sample size.
5. In a prospective study you can make the choice of most relevant and up to date outcome measures.

**DS-AN. Among given values of the areas under the curve (AUC), choose the one that would fit the test that distinguishes between healthy and ill participants the least:**

A. 1.0

B. 0.7

C. 0.5

D. 0.3

E. 0.0

**S4. You want to see what is the probability of getting colon cancer if you sit more than 10 hours a day for a long period of time.**

| **Answer** | **Hypothesis** | **Study design** | **Inclusion criteria** | **Exclusion criteria** | **Outcome measure** |
| --- | --- | --- | --- | --- | --- |
| A | What is the probability of getting colon cancer if you spend more than 10 hours daily sitting for a longer periods of time? | Systematic review of randomized controlled trials | Subjects who do not have colon cancer | Subjects with advanced stages of colon cancer | Colon cancer diagnosis |
| B | What is the probability of getting colon cancer if you spend more than 10 hours daily sitting for a long period of time? | Cohort study | Subjects who do not have colon cancer | Subjects who pass away during research | Incidence of colon cancer |
| C | What is the probability of getting colon cancer if you spend more than 10 hours daily sitting for a long period of time? | Randomized controlled trial | Subjects who have colon cancer | Subjects who do not want to participate | NNT (for sitting) |
| D | What is the probability of getting colon cancer if you spend more than 10 hours daily sitting for a long period of time? | Cross-sectional study | Subjects with and without colon cancer | Subjects who do not spend more than 10 hours a day sitting | Prevalence |
| E | Subjects who spend more than 10 hours daily sitting for a long period of time are more likely to get colon cancer | Case-control study | Subjects with and without colon cancer | Smokers | Odds ratio for getting colon cancer |

**CI-EV. Which of the following findings has the highest clinical value?**

1. Lowering blood cholesterol levels from 8 mmol/L to 6 mmol/L; P=0.049.
2. Lowering five-year mortality rate from prostate cancer for 7%; P=0.114.
3. Lowering mean arterial pressure from 111 mmHg to 107 mmHg; P<0.001.
4. Lowering five-year mortality rate from 5th year breast cancer for 2%; P=0.037.
5. Lowering blood cholesterol levels from 6 mmol/L to 4.2 mmol/L; P=0.052.

**DS-AP. The prevalence of a disease is 10%. The diagnostic test used for it has 80% specificity and 90% sensitivity. If you get a positive result, what is the likelihood of actually having the disease?**

A. Around 33%

B. Around 30%

C. Around 38%

D. Around 90%

E. Around 80%

**EB-AN. Complete the summary with a conclusion.**

*Introduction:* To investigate if acute fear can actually „freeze the blood in your veins“, researchers recruited 24 healthy volunteers (under 30 years).

*Methods:* Via random selection, 14 of the volunteers were sorted into a group that watched a horror movie before watching a documentary, and the remaining 10 volunteers watched the movies in reverse order. The movies were about 90 minutes long each and the volunteers watched them 7 days apart. Primary outcome was blood levels of coagulation factor VIII. Secondary outcome was participants’ own experience of fear while watching the movies.

*Results:* Results show that, after watching the horror movie, mean difference in perceived fear was 5.4 units (95% CI 4.7-6.1) on a visual analogue scale (VAS), and blood levels of coagulation factor VII were significantly increased (mean difference 11.1 IU/dL (111 IU/L), 95% CI 1.2-21.0 IU/dL).

***Conclusion* is the following:**

1. There was a statistically significant difference in levels of coagulation factor VIII after watching the documentary.
2. We cannot conclude with certainty, but the results indicate that horror movies increase blood levels of coagulation factor VII.
3. There is not enough evidence to conclude that horror movies affect blood levels of coagulation factor VIII.
4. Fear can actually „freeze the blood in your veins“.
5. There was a statistically significant increase of coagulation factor, but not of perceived fear.

**IS-AP. You want to find the article that described the study mentioned above. What combination of key words would you choose to find the exact same article?**

1. Healthy volunteers, documentary/horror movie, factor VIII.
2. Healthy volunteers, factor VIII, visual analogue scale, horror movie.
3. Healthy volunteers, horror movie, visual analogue scale, factor VIII.
4. Healthy volunteers, horror movie, documentary, factor VIII.
5. Horror movie, documentary, visual analogue scale, factor VIII.
